# Supplementary material for: Drivers of Under-Five Stunting Trend in 14 Low- and Middle-Income Countries since the Turn of the Millennium: A Multilevel Pooled Analysis of 50 Demographic and Health Surveys
Source: Nutrients. 2019 Oct 16;11(10):2485. doi: 10.3390/nu11102485 (PMC6835629; doi:10.3390/nu11102485)

Figure S1. Trends in indicators by country

A. Gini coefficient

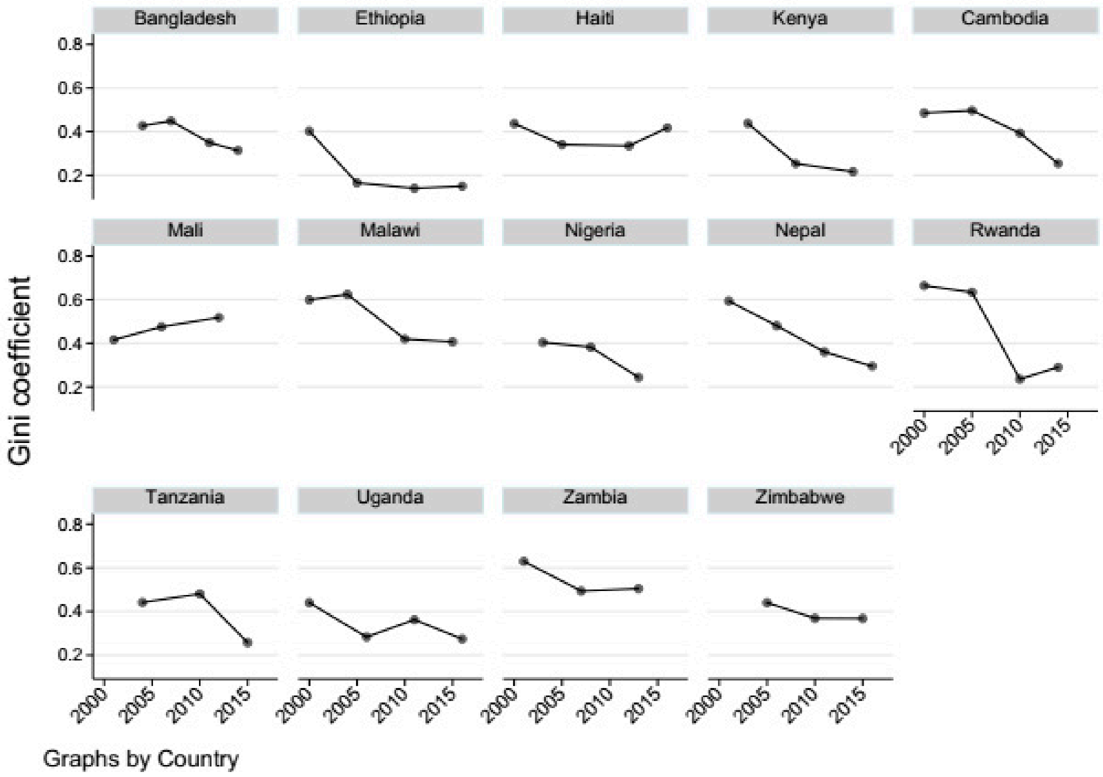

B. Total fertility rate

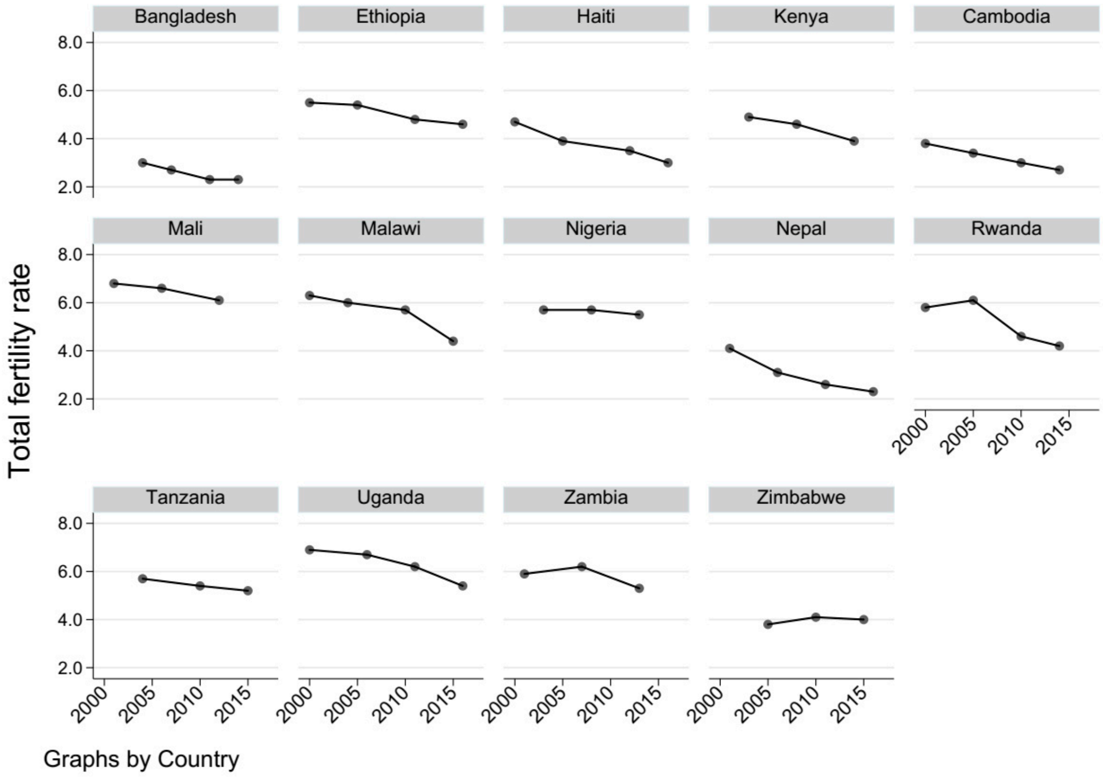

C. Urbanization

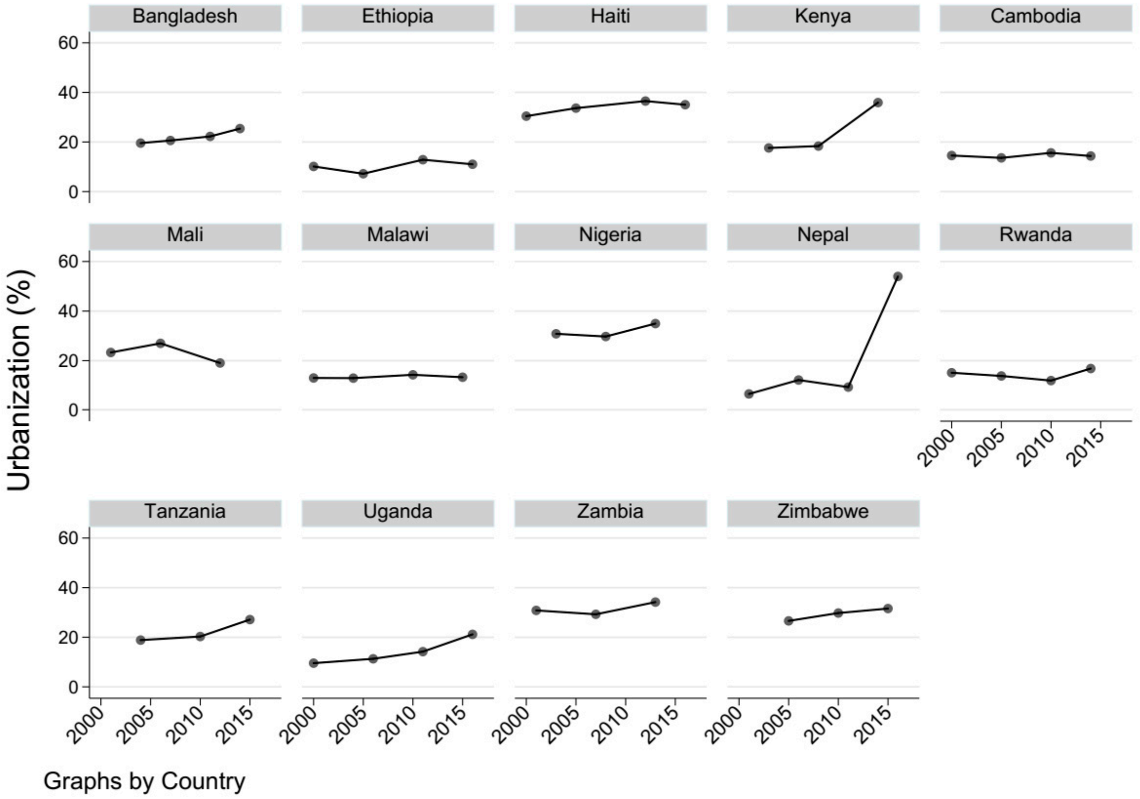

D. Female primary education

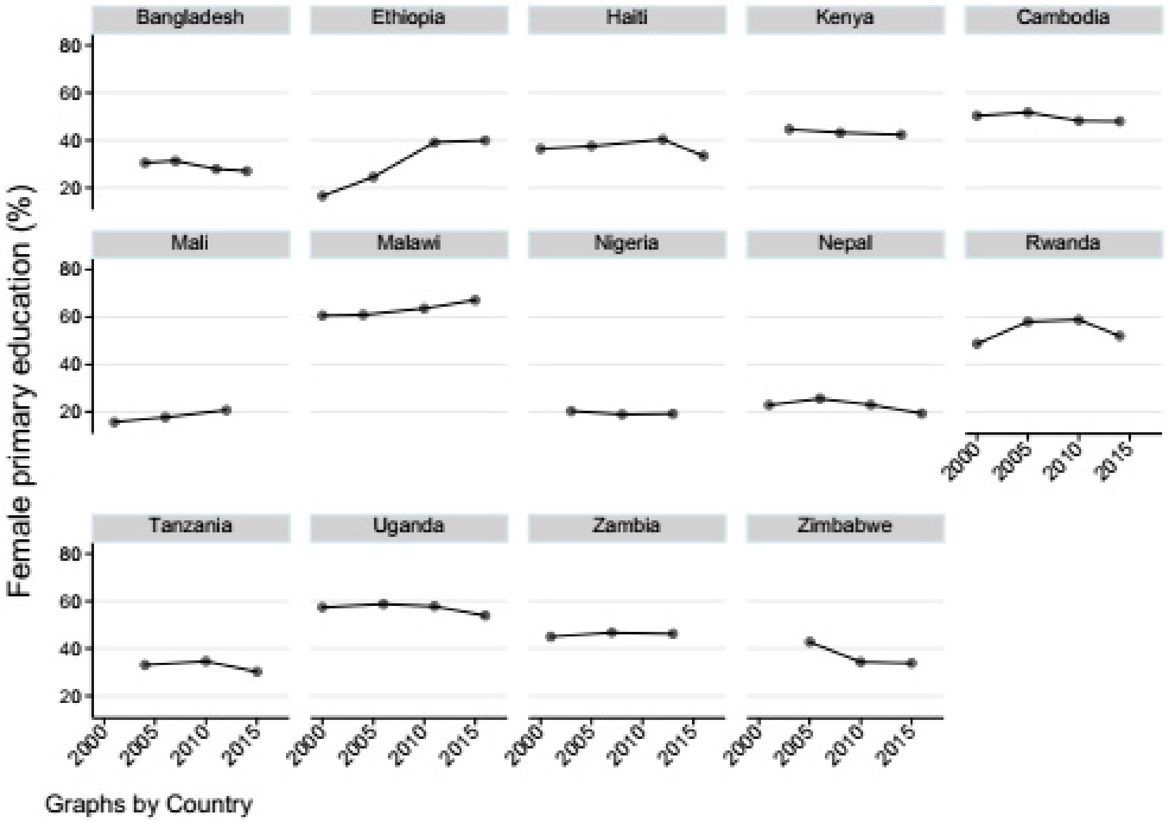

E. Male secondary education

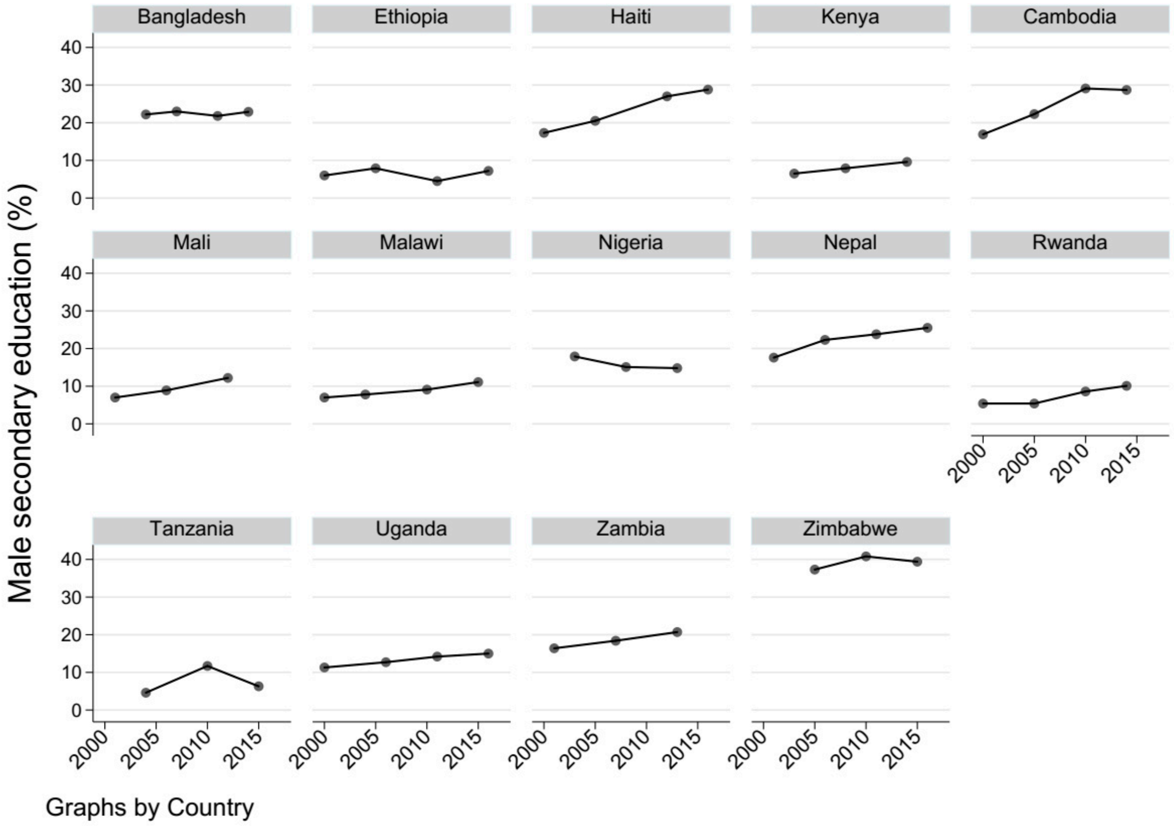

F. Women's decision -making power

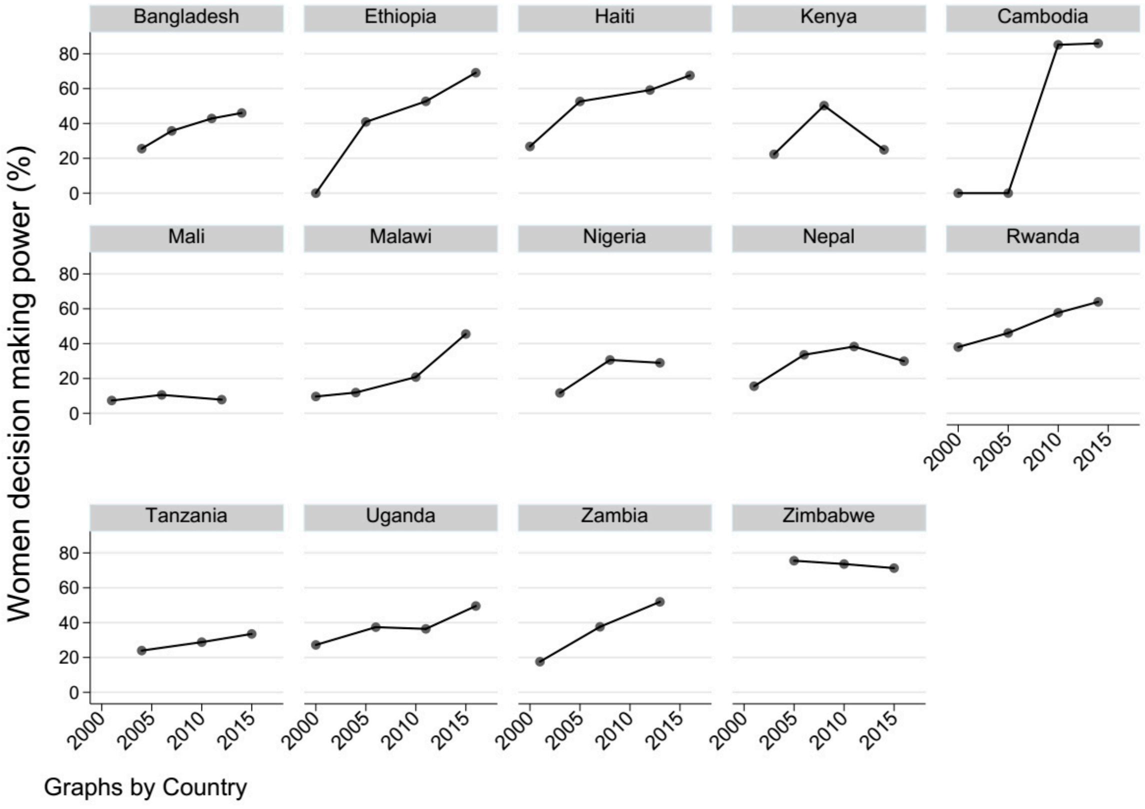

G. Women working

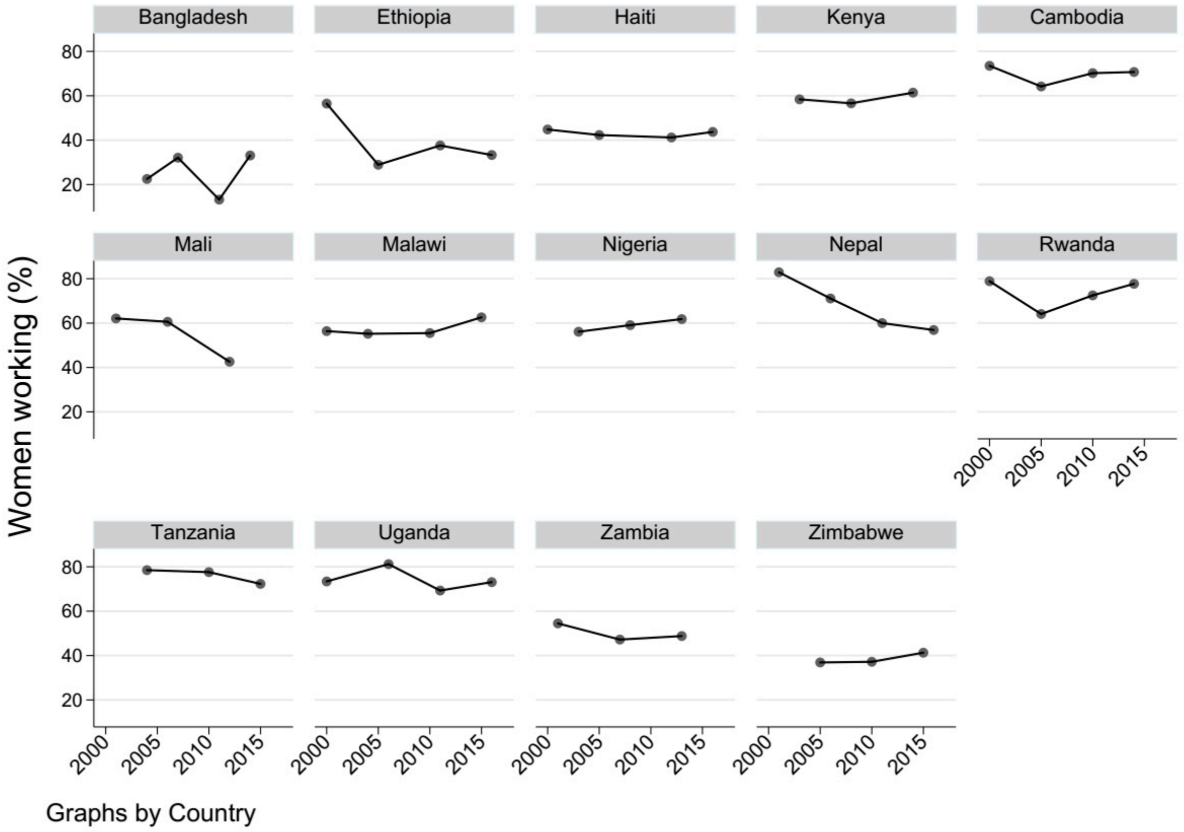

H. Improved sanitation facility

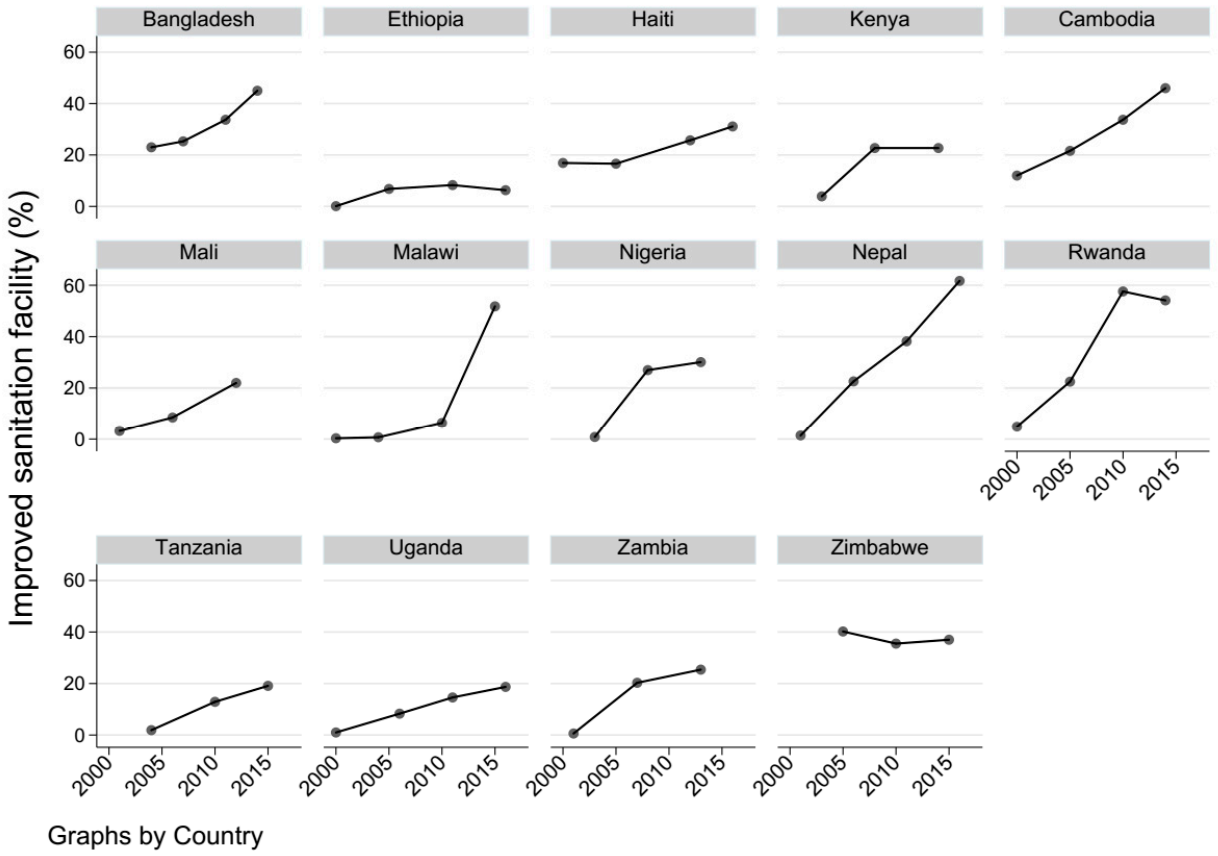

I. Improved drinking water source

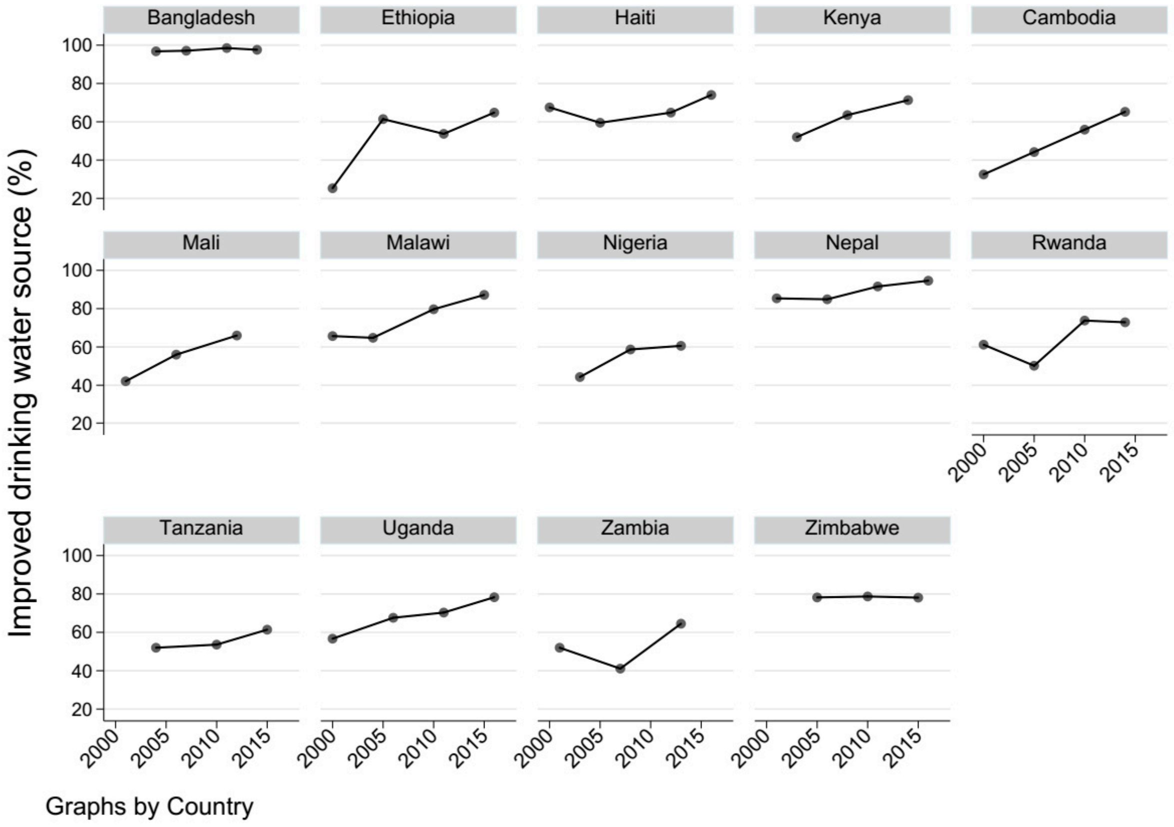

J. Antenatal care follow-up of 4+

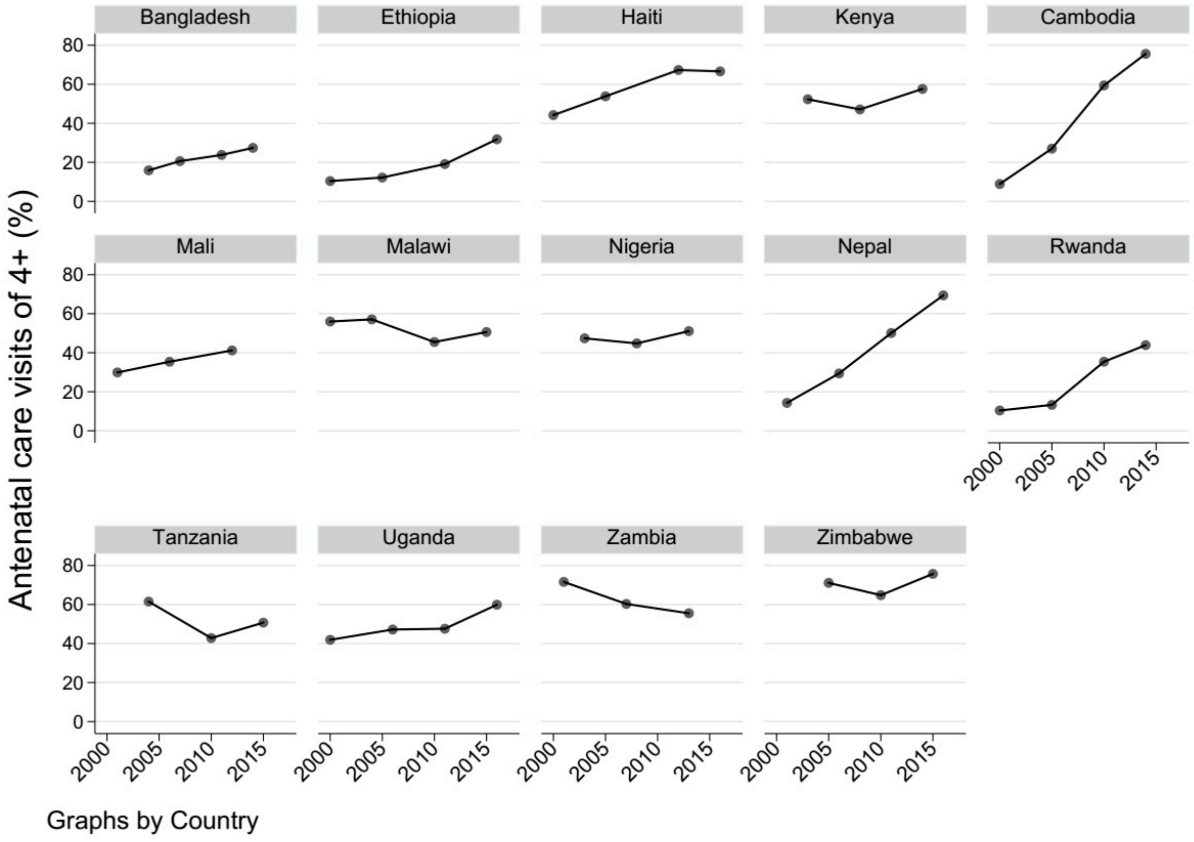

K. Delivery at health facility

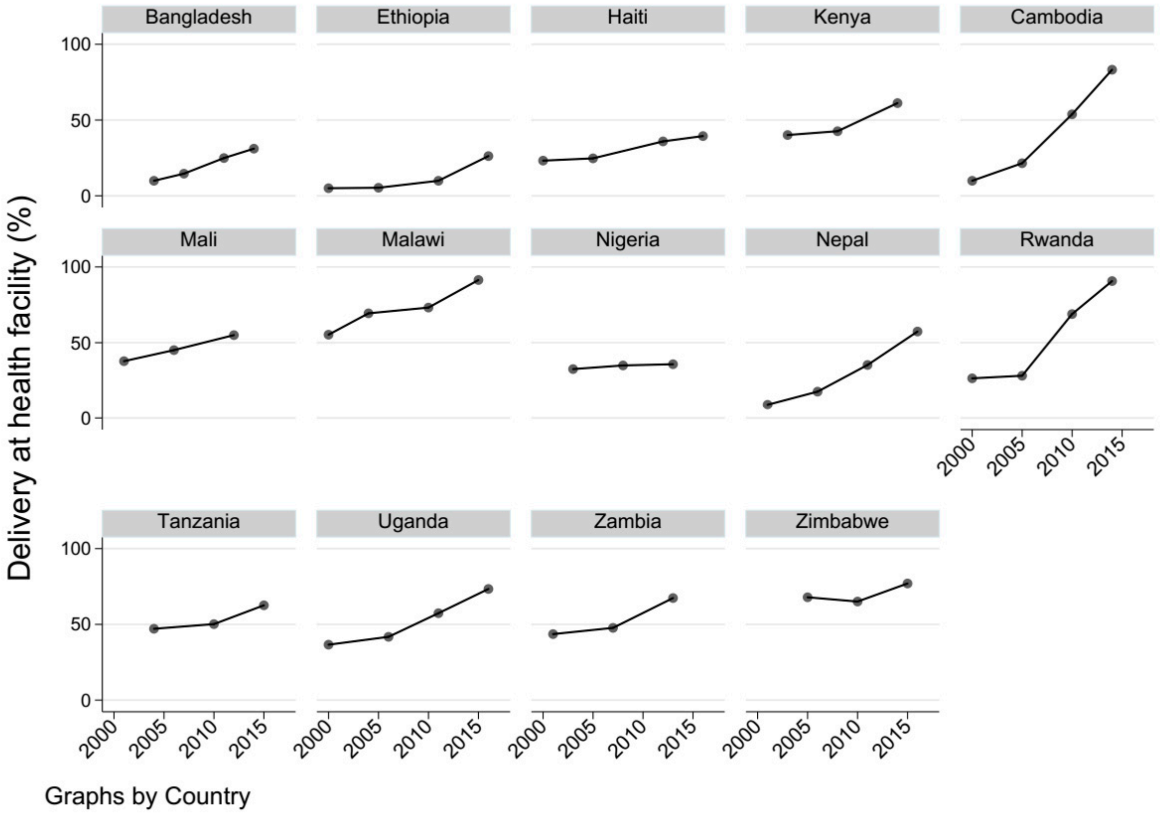

L. Iron supplementation during pregnancy

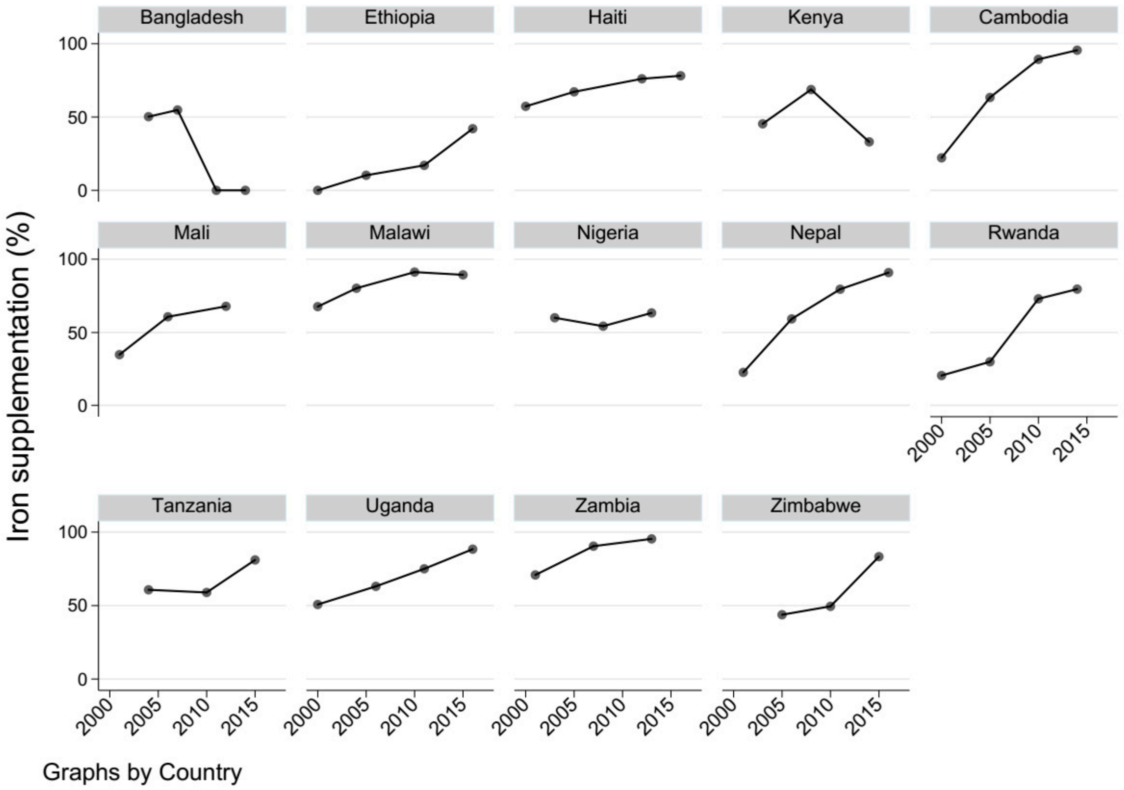

M. Children with basic vaccinations

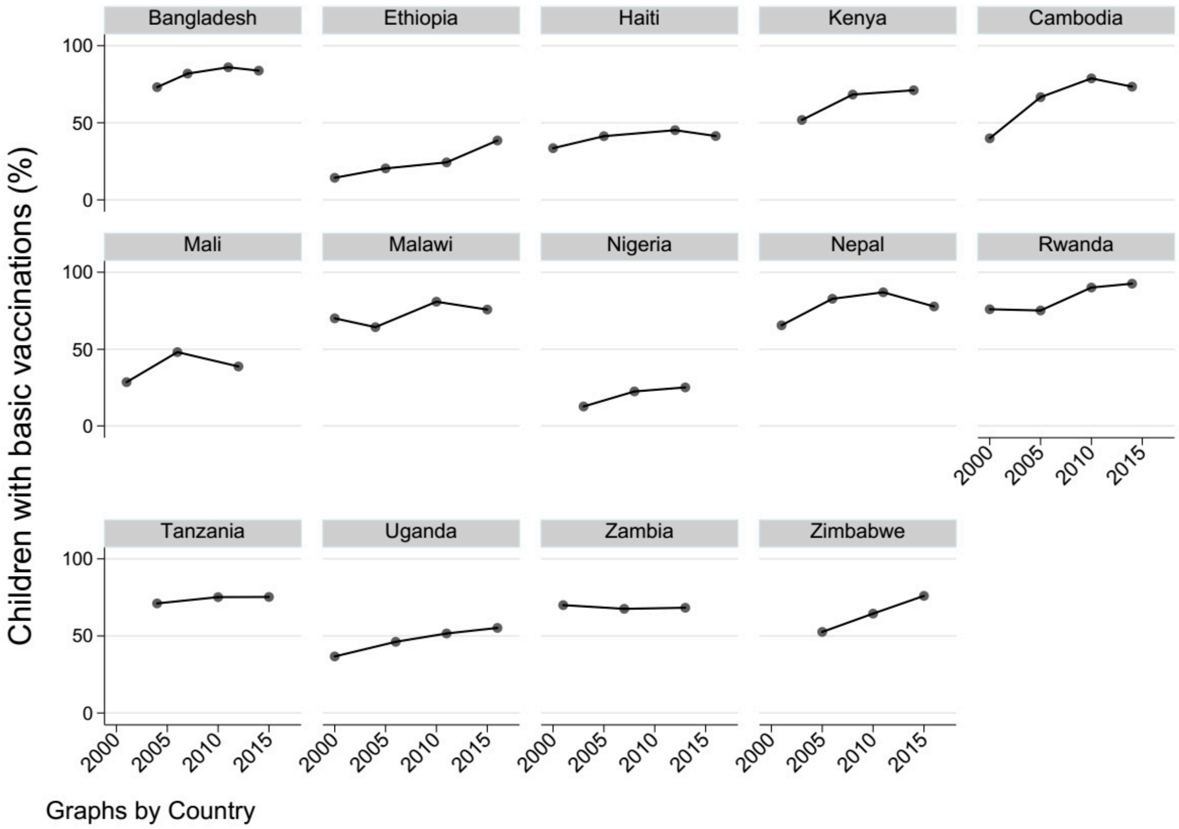

N. Breastfeeding initiation within ≤ 1 day

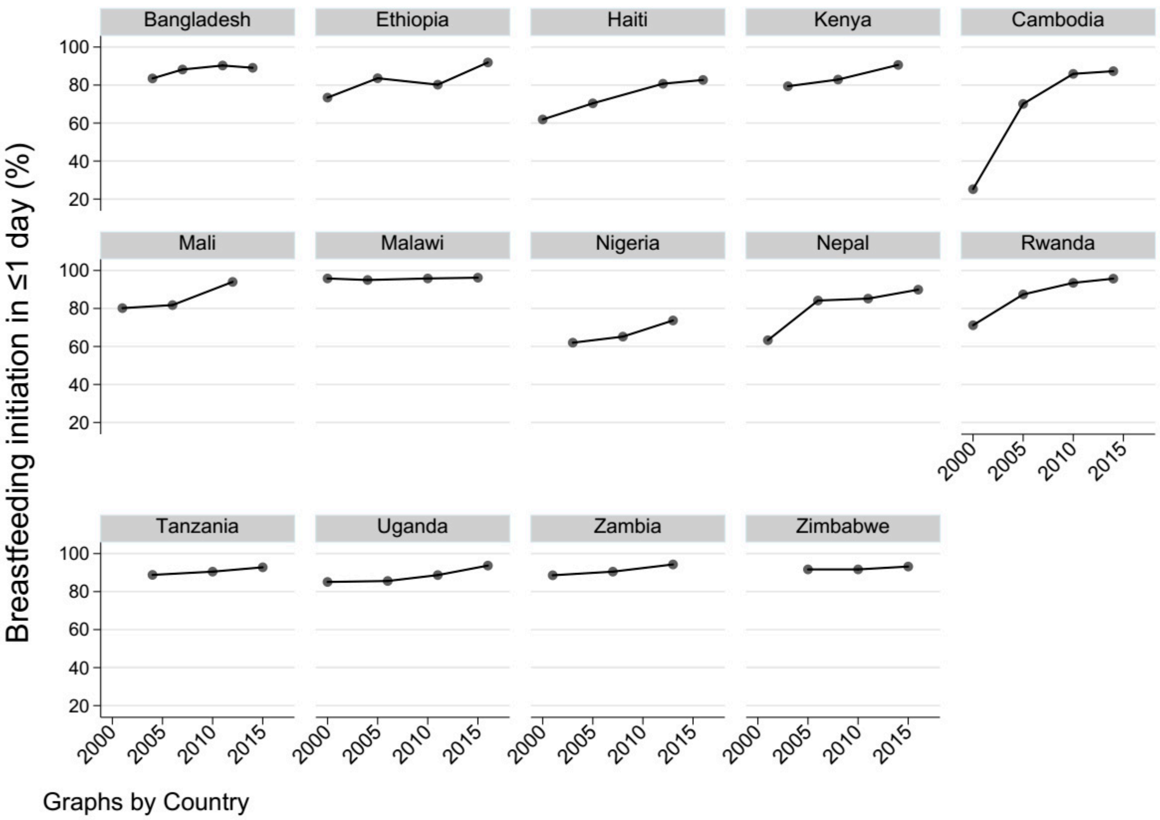

O. Median duration of exclusive breastfeeding

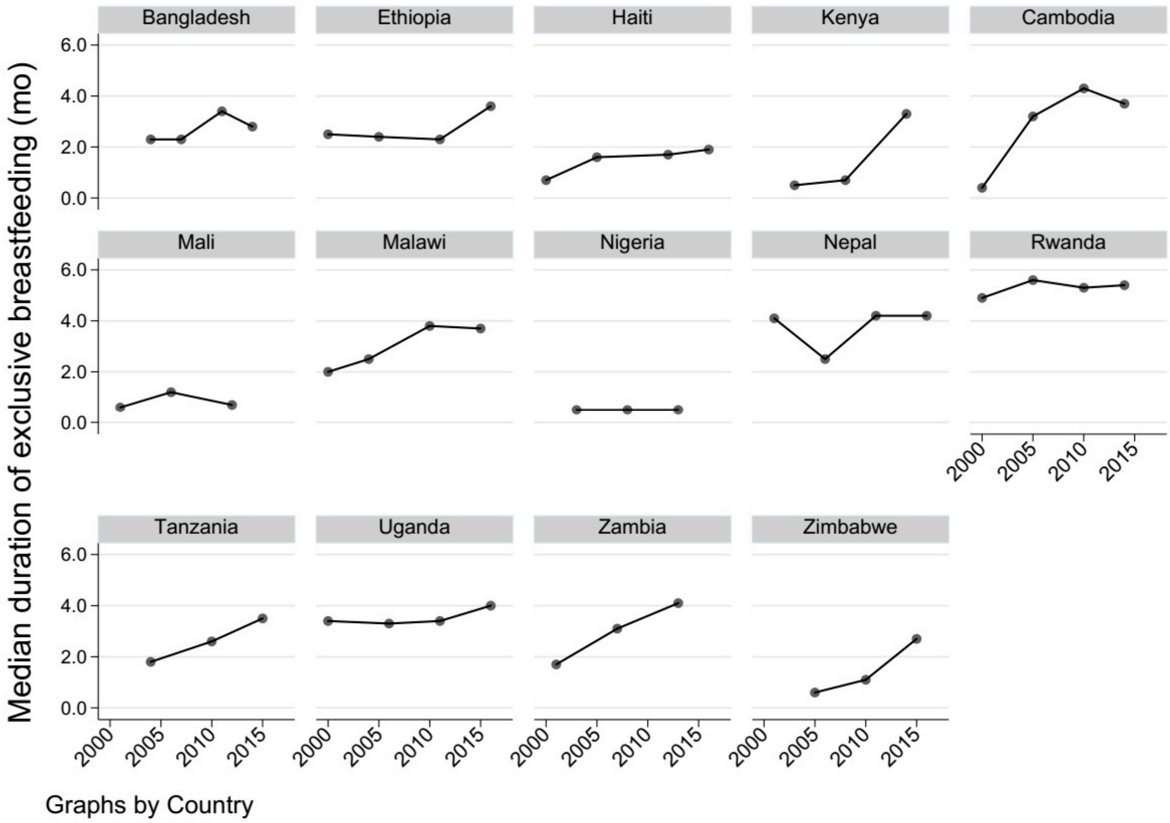

P. Complementary feeding 6-9 months age

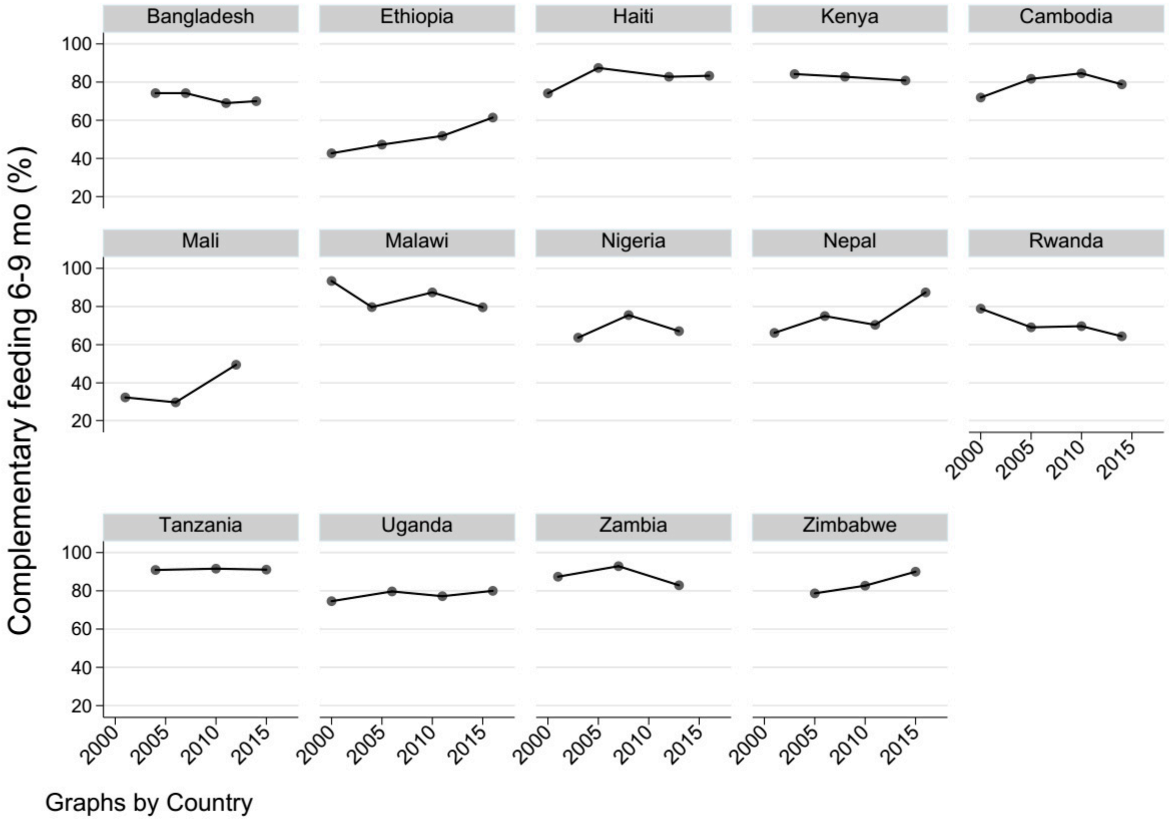

Q. Reported low birthweight

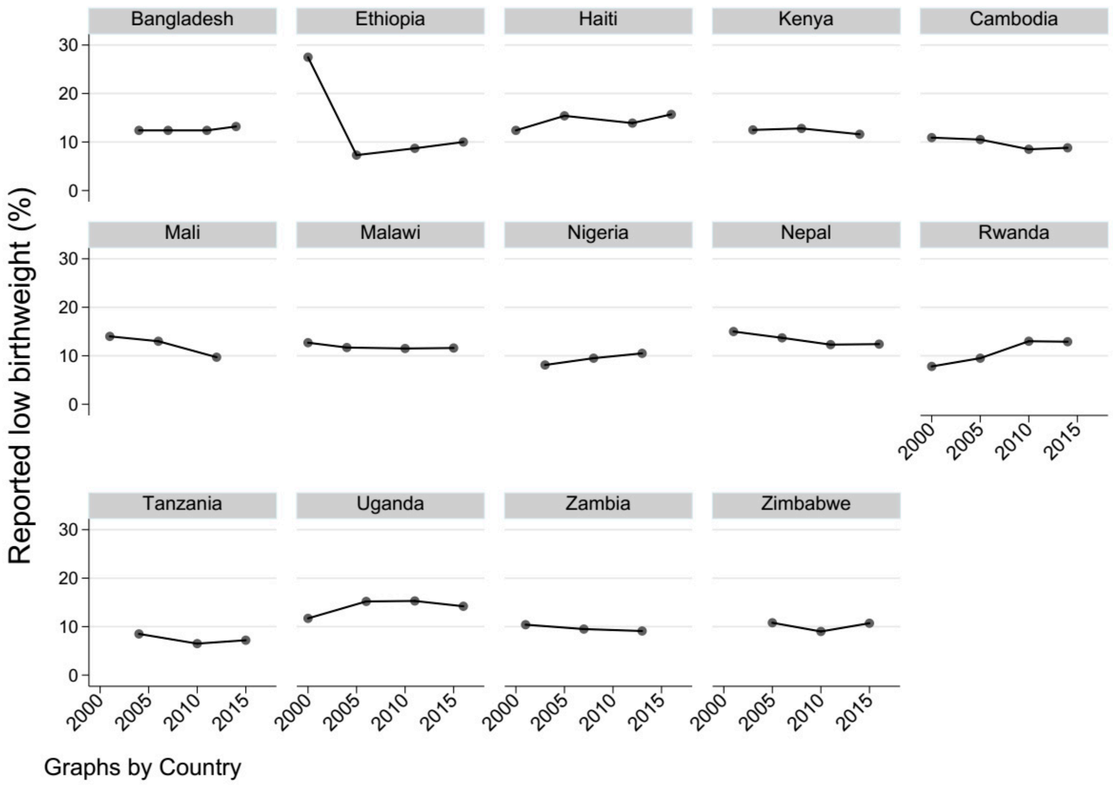

R. Prevalence of acute respiratory illness

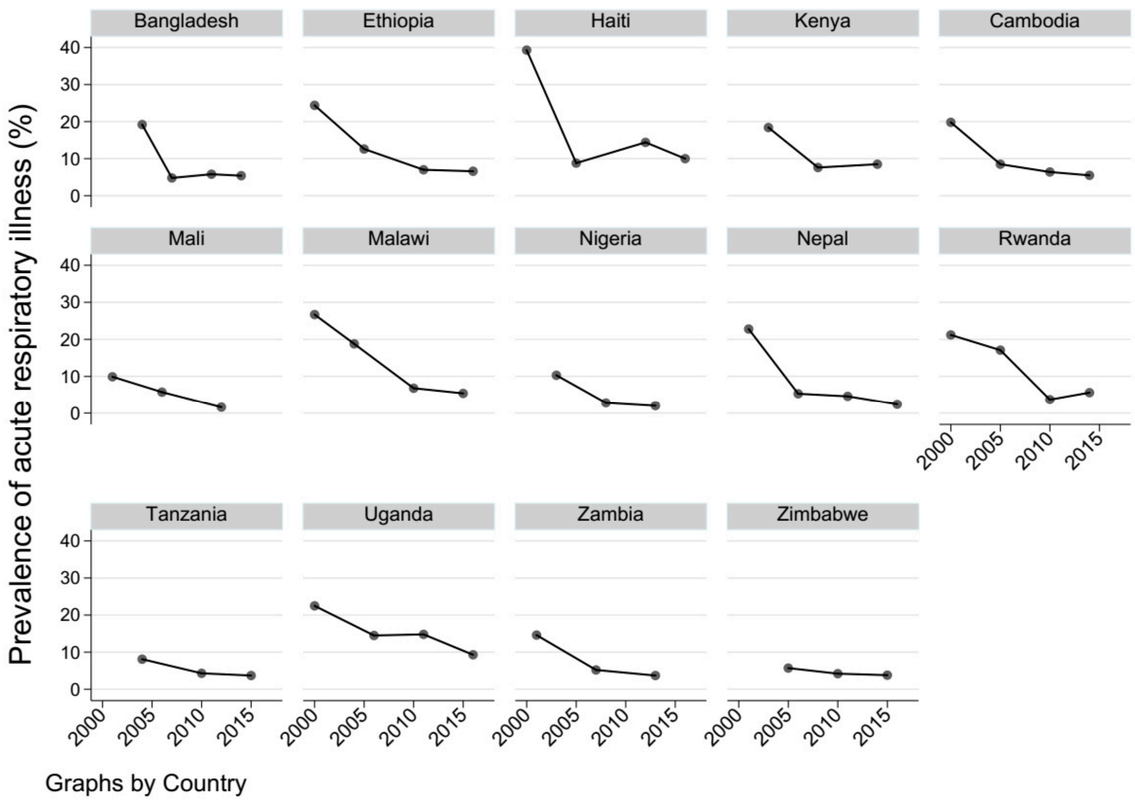

S. Prevalence of diarrhea

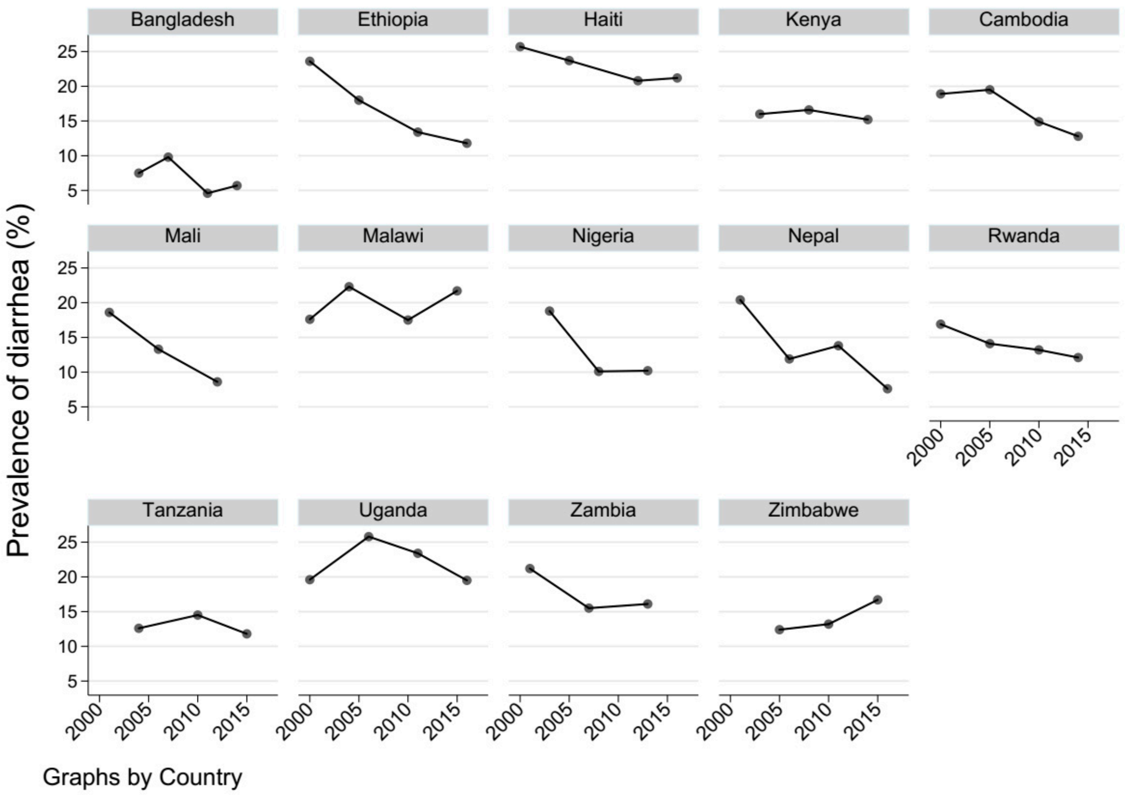

Supplement: Supplementary file 1 [file nutrients-11-02485-s001.pdf]
